# Alcohol Consumption and Common Carotid Intima-Media Thickness: The USE-IMT Study

**DOI:** 10.1093/alcalc/agx028

**Published:** 2017-05-19

**Authors:** Annie R. Britton, Diederick E. Grobbee, Hester M. den Ruijter, Todd J. Anderson, Moise Desvarieux, Gunnar Engström, Greg W. Evans, Bo Hedblad, Jussi Kauhanen, Sudhir Kurl, Eva M. Lonn, Ellisiv B. Mathiesen, Joseph F. Polak, Jacqueline F. Price, Christopher M. Rembold, Maria Rosvall, Tatjana Rundek, Jukka T. Salonen, Coen Stehouwer, Tomi-Pekka Tuomainen, Michiel L. Bots

**Affiliations:** 1 Department of Epidemiology and Public Health University College London, London WC1E 6BT, UK; 2 Julius Center for Health Sciences and Primary Care, University Medical Center Utrecht, 3508 GA Utrecht, The Netherlands; 3 Laboratory of Experimental Cardiology, University Medical Center Utrecht, 3508 GA Utrecht, The Netherlands; 4 Department of Cardiac Sciences and Libin Cardiovascular Institute of Alberta, University of Calgary, Calgary AB T2N, Canada; 5 Columbia University, 116th and Broadway, New York, NY 10027, USA; 6 Department of Clinical Sciences in Malmö, Lund University, Skane University Hospital, Jan Waldenströms gata 35, Malmö, Sweden; 7 Department of Biostatistical Sciences and Neurology, Wake Forest School of Medicine, Winston-Salem, NC 27157, USA; 8 Institute of Public Health and Clinical Nutrition, University of Eastern Finland, FI-70211 Kuopio, Finland; 9 Department of Medicine, Division of Cardiology and Population Health Research Institute, McMaster University, Hamilton, ON LSL 2X2, Ontario, Canada; 10 Brain and Circulation Research Group, Department of Clinical Medicine, University of Tromsö, N-9037 Tromsø, Norway; 11 Department of Radiology, Tufts University School of Medicine, 800 Washington St, Boston, MA 02111, USA; 12 Usher Institute of Population Health Sciences and Informatics, University of Edinburgh, EH16 4UX, UK; 13 Cardiology Division, Department of Internal Medicine, University of Virginia, Charlottesville, VA 22908-0158, USA; 14 Department of Neurology, Miller School of Medicine, University of Miami, Miami, FL 33136, USA; 15 MAS-Metabolic Analytical Services Oy, 00990 Helsinki, Finland; 16 Department of Internal Medicine and Cardiovascular Research Institute Maastricht, Maastricht University Medical Center, 6229 ER Maastricht, The Netherlands

## Abstract

**Aims:**

Epidemiological evidence indicates a protective effect of light to moderate alcohol consumption compared to non-drinking and heavy drinking. Although several mechanisms have been suggested, the effect of alcohol on atherosclerotic changes in vessel walls is unclear. Therefore, we explored the relationship between alcohol consumption and common carotid intima media thickness, a marker of early atherosclerosis in the general population.

**Methods:**

Individual participant data from eight cohorts, involving 37,494 individuals from the USE-IMT collaboration were used. Multilevel age and sex adjusted linear regression models were applied to estimate mean differences in common carotid intima-media thickness (CIMT) with alcohol consumption.

**Results:**

The mean age was 57.9 years (SD 8.6) and the mean CIMT was 0.75 mm (SD 0.177). About, 40.5% reported no alcohol consumed, and among those who drank, mean consumption was 13.3 g per day (SD 16.4). Those consuming no alcohol or a very small amount (<5 g per day) had significantly lower common CIMT values than those consuming >10 g per day, after adjusting for a range of confounding factors.

**Conclusion:**

In this large CIMT consortium, we did not find evidence to support a protective effect of alcohol on CIMT.

## INTRODUCTION

Alcohol consumption is one of the biggest public health challenges facing modern society. It is associated with a large range of health conditions and ranks as the fifth leading risk factor for disease and injury worldwide (Lim *et al*., 2013). The relationship between alcohol and cardiovascular disease (CVD) is complex. Epidemiological evidence indicates a protective effect of light to moderate alcohol consumption compared to non-drinking and an increased risk of cardiovascular events amongst heavier drinkers (Corrao *et al*., 2004; Silverwood *et al*., 2014). However, it has been argued that the protective effect observed in observational studies may in part be due to misclassification errors, reporting biases and residual confounding (Stockwell *et al*., 2012). It is, therefore, crucial to explore the biological mechanisms underlying the association between alcohol intake and CVD to help determine causality.

These mechanisms are not fully elucidated and are likely to be complex, involving both acute and chronic effects and beneficial and harmful outcomes (Mathews *et al*., 2015). Suggested chronic effects of alcohol on the vascular system include favourable changes in lipid profile and other cardiovascular biomarkers (Brien *et al*., 2011), as well as deleterious changes in arterial hypertension, peripheral artery disease and atherosclerosis (Fernández-Solà, 2015; Mathews *et al*., 2015). The effect of alcohol on atherosclerotic changes in vessel walls is disputed (Kim *et al*., 2014). Some studies have found evidence of a J-shaped (Schminke *et al*., 2005; Lee *et al*., 2009; Xie *et al*., 2011; Bauer *et al*., 2013) or positive relationship (Zyriax *et al*., 2010; Britton *et al*., 2016). Other studies report no association (Mowbray *et al*., 1997; Djoussé *et al*., 2002; Zureik *et al*., 2004) or an inverse linear trend (Lee *et al*., 2009). To address the relationship of alcohol consumption and carotid atherosclerosis in multiple populations, we used data from the USE-IMT consortium (Den Ruijter *et al*., 2012) that collected individual level carotid intima-media thickness (CIMT) and clinical data from multiple cohorts. We assessed CIMT as an intermediate phenotype of early atherosclerosis and a marker of subclinical organ damage that independently predicts vascular events (Lorenz *et al*., 2012).

## MATERIALS AND METHODS

### Study population

Baseline data were from the USE-IMT collaboration, an individual participant data meta-analysis established to determine the incremental value of CIMT in predicting cardiovascular events (Den Ruijter *et al*., 2012). Population-based prospective cohort studies conducted with data on cardiovascular risk factors, common CIMT, and follow-up for cardiovascular events were identified through systematic literature searches and expert recommendation. In the present analysis, we included 37,494 individuals from eight studies in North America and Europe of whom information on alcohol intake was available (Atherosclerosis Risk in Communities, Edinburgh Artery Study, Kuopio Ischemic Heart Disease Risk Factor Study, Malmo Diet and Cancer Study, Multi-Ethnic Study of Atherosclerosis, Northern Manhattan Study, Tromso Study, and Whitehall II Study).

### Variables

Current alcohol consumption was measured in the individual cohorts by self-report questionnaire. Information was harmonized into grams per day and categorized at 0 g; from 0 to 4 g; from 5 to 9 g, from 10 to 19 g; from 20 to 29 g and from 30 g or over.

Smoking status was ascertained from self-report questionnaires and defined as current, never or former smoking. For each individual, body mass index (BMI) was calculated from measured body weight (in kilograms) divided by measured height (in metres) squared. High blood pressure was defined as systolic blood pressure (SBP) ≥140 mmHg or diastolic blood pressure (DBP) ≥90 mmHg. Serum cholesterol was measured as total cholesterol and high-density lipoprotein (HDL) cholesterol levels. History of CVD and presence of diabetes mellitus were defined using the definitions of the individual cohorts, i.e. based on questionnaire information, and /or use of blood glucose lowering medication or fasting glucose level (den Ruijter *et al*., 2013).

### Statistical analysis

In order to standardize the common CIMT measurements, we rescaled CIMT levels using the method described by Engelen *et al*. (2013). We derived a linear regression model in the whole cohort with common CIMT as a function of cardiovascular risk factors (age, sex, blood pressure, diabetes, total cholesterol, HDL cholesterol, smoking and antihypertensive drug use) and a dummy variable for study. The reference study was the Atherosclerosis Risk in Communities Study (ARIC). Subsequently, for every individual in a cohort, we subtracted the measured CIMT with the regression coefficient of that cohort. In that sense, we adjusted for the variability between individuals that can be attributed to being in a certain cohort study, all relative to the ARIC results.

The relationship between alcohol consumption and common CIMT was evaluated using linear regression models, adjusting for age, sex, smoking, medical history and medication, blood pressure, BMI and serum cholesterol. Separate analyses were also run by sex.

Mean differences in common CIMT by increase in alcohol intake category were obtained with 95% confidence intervals (CIs), using the group with 10–19 g per day intake as reference group. This reference group was chosen in preference to the non-drinking group, as the latter consists of a mix of former drinkers and current abstainers. To study the relation between alcohol and more severe vascular abnormalities, we divided the population into those with a common CIMT >0.90 mm and those without.

## RESULTS

The mean age of the combined cohorts was 57.9 years (SD 8.6) and the mean CIMT was 0.75 mm (SD 0.177). Of the combined cohorts, 40.5% reported no alcohol consumed, and among those who drank, mean consumption was 13.3 g per day (SD 16.4).

**Fig. 1.**
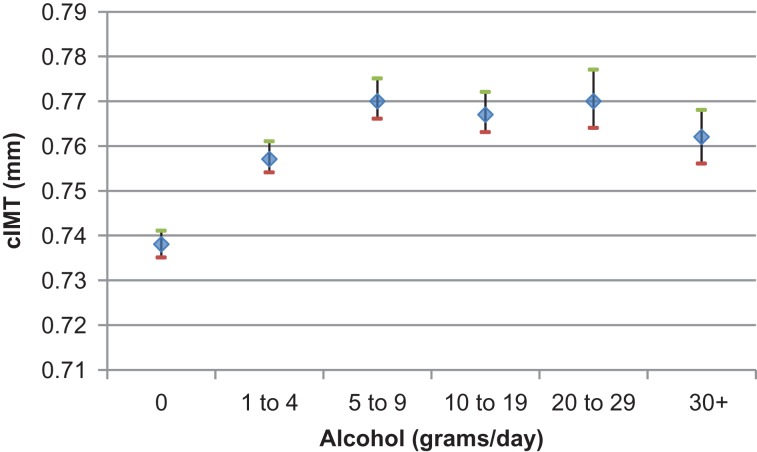
Relationship between alcohol consumption and common CIMT, adjusted for age, sex, medical history and medication, smoking, blood pressure, BMI and serum cholesterol.

There was no evidence of a J-shape or U-shaped relationship between alcohol consumption and CIMT (Fig. 1). In a fully adjusted model, compared to the reference group, i.e. those with an intake ranging from 10 to 19 g per day, those with no alcohol intake had significantly lower common CIMT values (mean difference −0.029 mm 95% CI −0.030; −0.024) and those with 1–4 g intake per day had a 0.01 mm lower common CIMT (Table 1). Those with the higher intakes than the reference groups had similar common CIMT values (Fig. 1). Similarly, a common CIMT above 0.90 mm was significantly less common in the no drinkers and lightest drinkers compared to the reference group. The results were similar when performed separately for men and women.

**Table 1.** Mean difference in CIMT (mm) by alcohol consumption group (reference 10–19 g per day)

Alcohol group (grams per day)	Adjusted mean difference	95% CI
0	−0.029	−0.030, −0.024
>0–4	−0.010	−0.016, −0.004
5–9	0.003	−0.003, 0.009
10–19		
20–29	0.003	−0.005, 0.011
30+	−0.005	−0.013, 0.002

Adjusted for age, sex, history CVD, SBP, DBP, hypertension medication, total cholesterol, HDL cholesterol, lipid lowering drugs, diabetes, BMI and smoking.

## DISCUSSION

We found no evidence of a protective effect of alcohol consumption on the arterial wall thickness assessed by an ultrasonic measure of common CIMT in a pooled study of 37,494 individuals from the general populations in Europe and the US. The lowest CIMT levels were reported for those who currently consumed no alcohol. Therefore, our findings do not support a protective vascular effect of moderate alcohol consumption that is mediated through an atherosclerotic pathway.

This is in agreement with other studies that found no marked relationship of alcohol and CIMT (Mowbray *et al*., 1997; Djoussé *et al*., 2002; Zureik *et al*., 2004) or a positive relationship (Mowbray *et al*., 1997; Djoussé *et al*., 2002; Zureik *et al*., 2004; Zyriax *et al*., 2010; Britton *et al*., 2016). Others have found a U-shaped relationship between alcohol and CIMT in cross-sectional analyses. For example, in the Cardiovascular Health Study investigating subjects over 65 years, consumers of 1–6 drinks per week (equalling <15 g/d) had a carotid IMT 0.07 mm lower than abstainers, whereas consumers of 14 or more drinks (equalling >30 g/d) had an IMT 0.07 mm higher than abstainers (Mukamal *et al*., 2003). The disagreement in findings requires further investigation. Possible explanations include different adjustments for confounders, patterns of consumption, types of beverages and different choices of reference groups.

The strength of this current study was our ability to harmonize across eight studies to pool data on >37,000 participants. This gave us sufficient power to detect differences in CIMT by drinking group and our results are generalizable across European and North American populations. However, the study is cross-sectional and we were not able to separate former drinkers from the current non-drinkers. Furthermore, we were not able to look at pattern of drinking or beverage type.

In conclusion, we do not find evidence to support a protective effect of alcohol on CIMT, a marker of subclinical CVD.
